# Investigating functional brain connectivity patterns associated with two hypnotic states

**DOI:** 10.3389/fnhum.2023.1286336

**Published:** 2023-12-19

**Authors:** Nuno M. P. de Matos, Philipp Staempfli, Erich Seifritz, Katrin Preller, Mike Bruegger

**Affiliations:** ^1^Clinic of Cranio-Maxillofacial and Oral Surgery, Center of Dental Medicine, University of Zurich, Zurich, Switzerland; ^2^MR-Center of the Department of Psychiatry, Psychotherapy and Psychosomatics, Department of Child and Adolescent Psychiatry, Psychiatric Hospital, University of Zurich, Zurich, Switzerland; ^3^Department of Psychiatry, Psychotherapy and Psychosomatics, Psychiatric Hospital, University of Zurich, Zurich, Switzerland

**Keywords:** distinct hypnosis states, functional connectivity, multi-voxel-pattern-analysis, physiological parameter, respiration, posterior hot zone, altered consciousness

## Abstract

While there’s been clinical success and growing research interest in hypnosis, neurobiological underpinnings induced by hypnosis remain unclear. In this fMRI study (which is part of a larger hypnosis project) with 50 hypnosis-experienced participants, we analyzed neural and physiological responses during two hypnosis states, comparing them to non-hypnotic control conditions and to each other. An unbiased whole-brain analysis (multi-voxel- pattern analysis, MVPA), pinpointed key neural hubs in parieto-occipital-temporal areas, cuneal/precuneal and occipital cortices, lingual gyri, and the occipital pole. Comparing directly both hypnotic states revealed depth-dependent connectivity changes, notably in left superior temporal/supramarginal gyri, cuneus, planum temporale, and lingual gyri. Multi-voxel- pattern analysis (MVPA) based seeds were implemented in a seed-to-voxel analysis unveiling region-specific increases and decreases in functional connectivity patterns. Physiologically, the respiration rate significantly slowed during hypnosis. Summarized, these findings foster fresh insights into hypnosis-induced functional connectivity changes and illuminate further knowledge related with the neurobiology of altered consciousness.

## 1 Introduction

Modern forms of hypnosis including hypnosis therapy are experiencing a real surge in popularity within a wide range of applications (Pekala, 2013, 2016; Wickramasekera, 2015; Zahedi and Sommer, 2021). According to the American Psychological Association (APA), hypnosis is defined as “A state of consciousness involving focused attention and reduced peripheral awareness characterized by an enhanced capacity for response to suggestion” (Elkins et al., 2015). There are different methods for hypnotizing a person. Spoken words with suggestible content are most commonly used for a hypnosis induction (Zahedi and Sommer, 2021). A hypnotized person, depending of the hypnotic depth, might experience the following changes: feelings of deep mental and physical relaxation, mental absorption, diminishing of judging and monitoring, suspension of time and localization orientation, and sometimes the experience of automatic or extra-volitional own (motor) responses (Price and Barrell, 2014; Landry et al., 2017; Varga et al., 2017; Zahedi and Sommer, 2021; Bicego et al., 2022). However, there is currently no overarching concept regarding the definition of a hypnotic state (Mazzoni et al., 2013; Hinterberger, 2015; Landry et al., 2017; Zahedi and Sommer, 2021). The literature usually describes a change in waking consciousness, light or deep trance, and sometimes a continuum of fluctuating trance states - that is, a state that is often rather unstable and elusive. Moreover, it is assumed that the neutral hypnotic state, i.e., the hypnotic state *per se* without any therapeutic or clinical intervention, is highly individual. As a result, there may be marked heterogeneity in perception and associated neurophysiological processes, which could be reflected in large data variance when investigated (McGeown et al., 2015; Jensen et al., 2017).

Although hypnosis has been studied extensively, understanding of hypnosis-associated neural effects is limited and thus crucial open questions remain regarding the identification of neuronal mechanisms underlying hypnosis-induction as well as the hypnotic state *per se* (Wickramasekera, 2015; Pekala, 2016; Landry et al., 2017).

Several key factors are repeatedly discussed as impeding the study of hypnosis according to scientific principles which require careful consideration (Barnier and Nash, 2012; Kihlstrom, 2013; Landry and Raz, 2015; Landry et al., 2017; Zahedi and Sommer, 2021). There are often (i) no coherent methodological standards and high variability in induction procedures, (ii) no adequate control conditions, (iii) no comparisons of hypnotic states inside versus outside the scanner, (iv) small number of study participants implying low statistical power, and (v) studies with unimodal approaches (mainly fMRI).

A great challenge is the standardization of hypnotic induction and design of appropriate control condition in the MR setting (Kihlstrom, 2013; Oakley and Halligan, 2013; Jensen et al., 2017; Landry et al., 2017; Varga et al., 2017; Zahedi and Sommer, 2021). An adequate control condition should match the semantic content of the hypnosis induction without the hypnagogic effect (Varga et al., 2017).

Based on all these methodological concerns and open questions we have developed a multi-study research project [fMRI, MR spectroscopy and electroencephalography (EEG)] not investigating therapeutic applications or interventions, but exclusively focusing on neural correlates that can distinguish hypnotic states from normal waking states of consciousness.

In this fMRI-study, brain connectivity patterns of two hypnotic states differing in depth (HS1 and HS2) are investigated by comparing them with two matching control conditions (CS1 and CS2). We employed the hypnosis induction procedure described by Dave Elman (Barth et al., 2019) in 50 healthy participants who were experienced in this technique. Functional connectivity was assessed by means of multivoxel pattern analysis, a purely data-driven approach without *a priori* assumptions (Whitfield-Gabrieli and Nieto-Castanon, 2012; Arnold Anteraper et al., 2019; Nieto-Castanon, 2020). Connectivity measures are complemented by respiration and pulse oximetry together with a short questionnaire.

Collectively, this study shows hypnosis-induced changes in the neural connectome and provides further indications of hypnotic depth-dependent connectivity differences as demonstrated by other groups (McGeown et al., 2015).

## 2 Materials and methods

### 2.1 Subjects

The study was approved by the Zurich cantonal ethics committee. All participants received detailed information about the experimental procedure, aim of the study, and provided their written informed consent before any procedure was performed. Participants were instructed not to consume alcohol, analgesics/other medication 24 h before the start of the experiment and to eat before arriving at the study site. The study was conducted at the MR center of the Psychiatric University Hospital Zurich, Switzerland.

**FIGURE 1:**
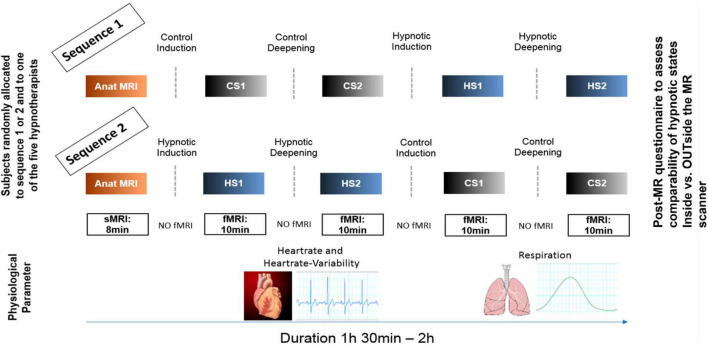
Experimental design. Participants were randomly allocated to two different experimental sequences to counterbalance sequential effects. Both sequences were identical except that for sequence 1, the control conditions (CS1, CS2) were performed first, followed by the hypnosis induction and deepening. In sequence 2, the order was reversed. During all fMRI measurements, heart rate and respiration data were recorded. In both sequences, a post-MR questionnaire was given to the participants to evaluate the comparability of the states compared to when under familiar circumstances (OUTside the scanner). Furthermore, the questionnaire also assessed stability of the states during the measurements, tiredness of the participants during the measurements and applied effort to maintain the states (including wakeful state during control conditions).

In total, 55 healthy participants (37 females, 18 males, mean age 46.9) were recruited and randomly allocated to either sequence 1 or 2 of the experimental procedure (Figure 1). Recruitment focused on participants familiar with the hypnosis procedures used in this study. Participants underwent basic hypnosis training (Hypnose.NET GmbH/OMNI Hypnosis International). All of them practice self-hypnosis on a weekly basis for at least 2°months. Their experience enabled them to remain in the hypnotic states during the fMRI measurement, despite the typical scanner setting. All were asked after the MR measurements whether the hypnotic experience inside the scanner was comparable to the experience they normally have and know outside the MR scanner.

### 2.2 Experimental design

Figure 1 illustrates the experimental design. In both sequences, first structural T1 weighted anatomical data were acquired. Sequence 1 then started with control state 1 (CS1) and control state 2 (CS2), followed by hypnosis state 1 (HS1) and hypnosis state 2 (HS2), whereas sequence 2 started with HS1 and HS2, followed by CS1 and CS2.

Each of these states was induced by spoken texts which were rigorously standardized, i.e., all participants heard identical texts. During the induction phases, no MR measurements were performed. To guarantee optimal speech quality for the induction texts and optimal noise reduction during MR measurements, specially designed headphones (MRConfon, Magdeburg, Germany) were used. The aim of the different hypnosis induction procedures was to induce different depth levels of neutral hypnosis. For induction into HS1, a Dave Elman induction, slightly adapted to the MR scanner environment and translated to German (Hypnose.NET GmbH/OMNI Hypnosis International), was used to induce a profound mental and physical relaxation (exact wording can be found in the Supplementary Appendix). HS1 was then further deepened in a second step, also based on a Dave Elman deepening method adapted to the MR scanner environment and translated to German, in order to achieve a deep state of hypnosis (HS2) which typically results in suspension of time and localization orientation and experience of automatic or extra-volitional own (motor) responses (Price and Barrell, 2014; Landry et al., 2017; Varga et al., 2017; Zahedi and Sommer, 2021; Bicego et al., 2022) (see Supplementary Appendix for exact wording).

The induction texts for HS1 and HS2 were counterbalanced by two control texts matching the semantic structures of both hypnosis texts as close as possible, but without the typical hypnotic-suggestive content. We constructed these control condition texts according to a recently published paper, discussing the importance of a valid control condition when investigating hypnosis on a basic scientific level (Varga et al., 2017). This was done by looking at the hypnosis texts paragraph by paragraph and taking similarly long Wikipedia entries with the main keywords. Control text 1 was semantically matched to induction text for HS1, and control text 2 was semantically matched to induction text for HS2, respectively, (see Supplementary material for exact wording of control text 1 and 2). For every participant, one out of five hypnosis experts was randomly allocated to read the induction texts.

After every text, the participants were instructed not to speak but to communicate only with raising their left index finger (as people tend to do tiny head movements when answering with yes or no) when they reached the corresponding state and were ready for the MR acquisition. This was monitored by a camera directed at the MR scanner bed.

Then, for every state - thus CS1/CS2 and HS1/HS2 - fMRI resting state data were acquired (sequence details see Figure 1). Furthermore, physiological parameters were assessed during the fMRI recordings by using the MR pulse-oximeter and the respiration belt. To optimize the comfortableness during the time spent within the scanner, we used the Philips Comfort Plus Mattress, Philips Healthcare, Best, The Netherlands.

### 2.3 MRI data acquisition

Data were acquired on a Philips Achieva 3T scanner (Philips Medical Systems, Best, The Netherlands), upgraded to the dStream platform, using a 32-channel receive-only head coil.

T1-weighted data were acquired with a 3D-T1w-TFE (Turbo-Field-Echo) sequence with following parameters: 160 sagittal slices, repetition time (TR) = 8.16 ms, echo time (TE) = 3.73 ms, acquisition voxel size = 1.0 × 1.0 × 1.0 mm^3^, flip angle = 8°, FOV = 240 × 240 × 160 mm^3^, acquisition matrix 240 × 240 pixels, scan duration 7 min 32 s.

For the fMRI resting-state data, identical sequences were used for all four experimental state conditions. A T2*-weighted echo planar imaging (EPI) sequence containing 220 volumes was applied with the following parameters: 45 axial slices, TR = 2500 ms, TE = 30 ms, in-plane acquisition voxel size = 3.00 × 3.00 mm^2^, slice thickness = 3.0 mm, flip angle = 78°, field of view (FOV) = 220 × 220 mm^2^, acquisition and reconstruction matrix = 128 × 128, SENSE = 1.8, slice gap = 0 mm. The acquisition of the functional volumes was preceded by five dummy scans not stored in the dataset, resulting in a total acquisition time of approximately 10 min per scan (Figure 1).

### 2.4 Analysis of fMRI data

#### 2.4.1 Step 1: Pre-Processing

Preprocessing and connectivity analyses were performed in MATLAB V2018b using the CONN toolbox V18.b (Whitfield-Gabrieli and Nieto-Castanon, 2012; Nieto-Castanon, 2022). For preprocessing, CONN uses functions from the SPM12 software V6906.^1^ We applied the standard preprocessing steps as suggested by (Whitfield-Gabrieli and Nieto-Castanon, 2012):

(1) Translation of the anterior commissure to the (0,0,0 mm) coordinates
(2) Realignment and unwarping
(3) Slice-timing correction
(4) Outlier detection using the Artifact Detection Tools implemented in CONN
(5) Co-registration of the functional image to the individual anatomical image (T1)
(6) Segmentation and normalization of the T1-image to the Montreal Neurological Institute (MNI) space
(7) Normalization of the functional images using the deformation matrix created in the previous segmentation and normalization of the T1-image
(8) Interpolation to a voxel size of 2°mm^3^
(9) Spatial smoothing with a Gaussian kernel with 8°mm full width at half maximum (FWMH).

#### 2.4.2 Step 2: Denoising and Filtering

Functional connectivity analysis needs more conservative approaches – compared to task-based fMRI - to appropriately control confounding influences of subject motion and other non-neuronal sources like e.g., pulsation or breathing artefacts (Birn et al., 2006; Van Dijk et al., 2010, 2012; Power et al., 2012). Thus, to remove such confounding effects we applied the CompCor method implemented in CONN (Behzadi et al., 2007). CompCor estimates the principal components (PCA) from the BOLD time series within white matter, cerebrospinal fluid (CSF) and large vessels, as BOLD time series alterations (in these so called “noise ROIs”) are unlikely to be modulated by neural activity. Removing these confounding components increases the signal to noise ratio by considering the derived PCAs as covariates in a general linear model (GLM) and enhances the detection of true effects evoked by the experimental conditions and thus a valid identification of correlated and anti-correlated networks (Behzadi et al., 2007; Whitfield-Gabrieli and Nieto-Castanon, 2012; Brauchli et al., 2019). We opted for the following confound dimensions: WM and CSF: Inf, without temporal and polynomial expansion; Realignment: Inf, with 1st-order derivatives and no polynomial expansion; Scrubbing: Inf, no temporal/polynomial expansion. This conservative approach allows the algorithm to reliably partialize out all confounding signals that are not subject to neural activation. The residual BOLD time series were then band-pass filtered from 0.01 to 0.1°Hz (after regression RegBP) and linearly detrended (Whitfield-Gabrieli and Nieto-Castanon, 2012).

After step 2, we very carefully inspected the data using the quality assessment options from the CONN toolbox. Based on this thorough inspection, four subjects were excluded from further analysis steps due to large movements or signal fluctuations.

#### 2.4.3 Step 3: Connectome Analysis

We opted for a purely data-driven multi-voxel- pattern analysis (MVPA) (Whitfield-Gabrieli and Nieto-Castanon, 2012; Nieto-Castanon, 2020, 2022) to classify the main inherent connectivity features. MVPA measure is an empirical method (completely inherent information based) to fully represent the shape of these connectivity patterns and/or some of their strongest features. The method identifies multivariate patterns of pairwise connections between all voxels in the brain (1st-level voxel-to-voxel covariance) and accounts for multivariate dependencies in the data, in contrast to standard univariate analysis that consider the effects of each voxel or cluster separately.

Such a strategy allows the model-free detection of areas/clusters fundamentally involved in the corresponding neuronal coding of the four conditions (HS1, HS2, CS1, and CS2).

The first level analysis was conducted with 10 PCA factors (principal component analysis), with a dimensionality reduction of 64 reflecting the standard approach using the CONN toolbox detailed in Whitfield-Gabrieli and Nieto-Castanon (2012), Nieto-Castanon (2022). The derived clusters were then further processed in a 2nd-level MVPA as described below.

##### 2.4.3.1 2nd-level analysis

Based on the test hypothesis that HS1 and HS2 represent two different hypnosis levels, means that the neural connectome supposedly differs between these conditions. Hence, the correct approach is to compare HS1 with the content-matched control condition CS1 and HS2 accordingly with CS2. A further important step is the comparison between the two control conditions. Here we assume that there are no differences in the MVPA-based functional connectivity architecture as both control texts transmit no hypnotic elements. The last step is the MVPA-based comparison between HS1 and HS2, as we hypothesize different neural connectomes between these two states. A total of four statistical analyses were conducted (CS1 vs. HS1; CS2 vs. HS2; HS1 vs. HS2, and CS1 vs. CS2). In order to account for multiple comparisons, the *p*-threshold of voxel height was set to 0.00025 (uncorrected) by dividing the typically applied threshold of 0.001 by 4. On cluster level, a false-discovery rate (FDR) correction with a p-level of 0.05 was applied (see Table 1).

**TABLE 1:** Applied contrasts and statistical significance thresholds in the 2nd-level MVPA analysis and *post-hoc* seed-to-voxel explorations.

Contrast	MVPA analysis		*Post-hoc* seed-to-voxel analysis		
	Height thresh. (uncorr.)	Cluster-level (FDR-corr)	Number of seed clusters	Height thresh. (uncorr.)	Cluster-level (FDR-corr)
CS1 vs. CS2	*p* = 0.00025	*p* = 0.05	No sig clusters from MVPA		
CS1 vs. HS1	*p* = 0.00025	*p* = 0.05	5	*p* = 0.00002	*p* = 0.05
CS2 vs. HS2	*p* = 0.00025	*p* = 0.05	4	*p* = 0.00025	*p* = 0.05
HS1 vs. HS2	*p* = 0.00025	*p* = 0.05	6	*p* = 0.000167	*p* = 0.05

FDR, False discovery rate; MVPA, Multi-voxel pattern analysis.

#### 2.4.4 Step 4: Seed-to-Voxel Analysis

The resulting clusters from the MVPA-analyses were used as seed ROIs to calculate a seed-to-voxel analysis in order to further explore the connectivity patterns underpinning the MVPA findings (Arnold Anteraper et al., 2019; Nieto-Castanon, 2022). The seed-to-voxel analyses were also performed using the CONN toolbox by calculating the bivariate correlations from average signal fluctuations from each cluster to all other voxels. The statistical thresholds were defined analogously to the MVPA analyses by bonferroni correcting the height threshold of *p* = 0.001 for the number of seed clusters identified for a given contrast in the MVPA analyses (height threshold contrast: *p* = 0.001/N clusters; cluster-level FDR-corr *p* = 0.05). Statistical thresholds are summarized in Table 1.

### 2.5 Psychophysiological data acquisition and analysis

Respiration and heart rate data were recorded during each rs-fMRI acquisition in order to assess potential psychophysiological effects associated with the hypnosis states. The MR-inherent respiration belt and the pulse oximeter were used for data acquisition and data was sampled at 496 Hz (Figure 1). Scanphyslog text files which were created for all rs-fMRI sessions were imported into LabChart Pro v8.1.2 (ADInstruments, Sydney, Australia) for analysis.

#### 2.5.1 Heart rate measures

Heart rate (HR) and Heart rate variability (HRV) were calculated using the HRV module integrated in the LabChart software. This module provides automated identification of the peaks in the pulse-oximetry signal and automatically provides HRV-estimates in the time and frequency domain. The pulse-oximetry signal was visually inspected for peak classification errors and manually corrected if necessary (de Matos et al., 2019).

HR was quantified as mean beats per minute during each of the measurements. HRV was calculated as the ratio of low frequency to high-frequency (LF/HF) components in the heart rate variability signal. The LH/FH-ratio has been suggested to reflect the ratio of sympathetic (LF) to parasympathetic (HF) activity levels of the autonomous nervous system (ANS). The higher the value, the stronger the relative sympathetic activity level (Cacioppo et al., 2007).

#### 2.5.2 Respiration

First, the respiratory signal was low-pass filtered at 0.5 Hz to remove high-frequency noise. Second, the peaks of each respiratory cycle (maximal inhalation) were identified using the peak analysis toolbox included in the LabChart software package for estimation and extraction of respiratory cycle lengths. Data was visually checked for classification errors and erroneous periods were excluded.

### 2.6 Questionnaires

After the MR experiment, as mentioned above, a specifically designed short questionnaire was conducted to assess qualitative behavioral aspects regarding the hypnosis experience during the MR measurements inside the scanner compared to the well-known experience outside the scanner.

### 2.7 Statistical analysis of psychophysiological and questionnaire data

Statistical analyses of heart rate, respiratory and questionnaire data were performed using SPSS 25 (IBM Corp., Armonk, NY, USA). A statistical threshold of *p* < 0.05 (two-tailed) was applied as significance criterion. First, the data was checked for its normal distribution by means of a Kolmogorov-Smirnov test of normality.

Depending on the test for normality distribution, comparisons of the four conditions were (1) either analyzed by means of an 2 × 2 factor analysis of variance (ANOVA) with the factors intervention (hypnosis, control) and depth (states 1 or 2), or (2) were compared using a non-parametric Friedman-test. In case of significances, *post-hoc* analyses were performed by means of Wilcoxon signed-rank test.

## 3 Results

The number of datasets included in the analyses for the different outcomes are summarized in Table 2.

**TABLE 2:** Overview of the number of data sets included in the analyses for the different outcomes.

fMRI-Study		
Analysis	Datasets analyzed	Reasons for exclusion
**Primary Outcome:**		
fMRI	50	5 datasets were excluded due to strong movement and/or excessive levels of global signal fluctuations
**Secondary Outcomes:**		
Questionnaires	50	
Respiration	44	6 additional datasets were excluded due to significant signal loss and artifacts
Heart rate and Heart rate variability	42	8 additional datasets were excluded due to significant signal loss and artifacts

Note that for the secondary outcomes only the 50 participants included in the primary outcome analyses were considered.

### 3.1 Behavioral aspects: hypnotic state characteristics and fidelity

Descriptive statistics from the questionnaire provided by the participants in order to assess quality and characteristics of the different states is listed in Table 3.

**TABLE 3:** Descriptives from the hypnotic state quality questionnaire.

Item	Mean (SD)			
	CS1	CS2	HS1	HS2
How comparable were the hypnotic states to those you know form OUTside the MR scanner? (1 = not at all/10 = identical)	−	−	8.67 (1.48)	8.36 (1.76)
Did the state quality change across the measurement? (1 = not at all/10 = changed completely)	2.32 (1.82)	2.38 (1.86)	2.56 (1.84)	3.27 (2.33)
Was it difficult to remain within the states? (1 = not at all/10 = very difficult)	2.88 (2.13)	3.02 (2.17)	1.88 (1.62)	1.73 (1.19)
How close were you to fall asleep? (1 = not at all/10 = fell asleep)	1.98 (1.83)	2.12 (1.98)	1.35 (1.07)	1.37 (1.24)

CS1/CS2 indicates Control States, HS1/HS2 indicates Hypnotic States, SD, standard deviation.

### 3.2 Physiological results

The data from the physiological parameters including mean values and corresponding standard deviations is summarized in Table 4.

**TABLE 4:** Physiological Parameter measured in both studies.

Parameter	Mean (SD)			
	CS 1	CS 2	HS 1	HS 2
Heart Rate Variability (HRV) (Low Frequency/High Frequency Ratio)	1.11 (0.99)	1.14 (0.81)	1.38 (1.42)	1.39 (1.21)
Heart Rate (HR) (Beats per Minute)	66.74 (10.99)	66.59 (11.01)	66.54 (12.11)	67.09 (12.08)
Respiration (Amplitude peak-to-peak in seconds)	5.29 (2.73)	5.34 (2.76)	6.26 (3.47)	7.17 (5.07)

CS1/CS2 indicates Control States, HS1/HS2 indicates Hypnotic States, SD indicates standard deviation.

#### 3.2.1 HR and HRV

Analysis of the data HR and HRV-data found no significant differences between the four experimental conditions.

#### 3.2.2 Respiration

Due to significant deviations from normal distribution, the four conditions were simultaneously compared by means of a Friedman-test. It revealed significant differences between the conditions (X^2^ = 25.391, *p* < 0.001. *Post-Hoc* analyses by means of Wilcoxon-tests found significantly slower respiration frequencies during HS1 compared to CS1 (*Z* = −3.361, *p* = 0.001). Between CS2 and HS2, the average drop in respiration rate was even more pronounced with a mean of 7.17 s for a respiratory cycle also reaching statistical significance (*Z* = −2.778, *p* = 0.005).

The comparison of both hypnosis states (HS1, CS1) and control states (HS2, CS2) did not reach statistical significance for mean respiration rate.

### 3.3 fMRI results

**FIGURE 2:**
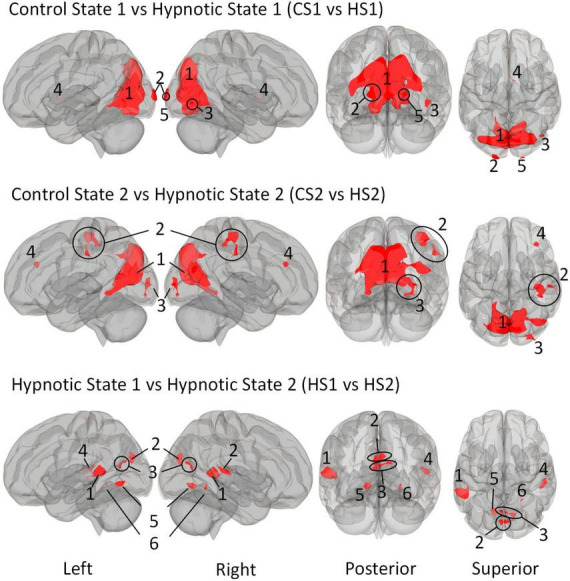
Illustration of the identified clusters using the Multi-voxel Pattern Analysis (MVPA) method for the three comparative analyses of the hypnotic states 1 and 2 (HS1 and HS2) and corresponding control states 1 and 2 (CS1 and CS2). The fourth comparison CS1 vs. CS2 did not reveal any clusters and is therefore not shown. Voxel height threshold of *p* < 0.00025 (Bonferroni corrected for the four performed MVPA analyses: *p* = 0.001/4) and cluster size threshold of *p* < 0.05 FDR were used as statistical thresholds.

We first describe the multi-voxel-pattern-analysis (MVPA) results for the comparisions control state 1 vs. hypnosis state 1 (CS1 vs. HS1), control state 2 vs. hypnosis state 2 (CS2 vs. HS2) and hypnosis state 1 vs. hypnosis state 2 HS1 vs. HS2), illustrated in Figure 2. The corresponding statistical details are summarized in Table 5 (CS1 vs. HS1), Table 6 (CS2 vs. HS2) and Table 7 (HS1 vs. HS2).

**TABLE 5:** Identified clusters from the Multi-Voxel Pattern Analysis (MVPA) for the contrast CS1 vs. HS1.

Cluster	Coordinates (x,y,z)			k	p-FDR	p-unc
1	10	−78	38	5931	<0.000001	<0.000001
2	−18	−106	2	130	0.000008	<0.000001
3	42	−74	−8	68	0.000907	0.00008
4	0	4	0	43	0.008153	0.000959
5	20	−104	4	37	0.012651	0.001861
**Anatomical coverage**						
	**Voxel count**		**% coverage**	**Region**		
Cluster 1	672		89	Intracalcarine cortex right		
	599		12	Lateral Occipital cortex right		
	576		38	Lingual gyrus left		
	569		33	Lingual gyrus right		
	500		78	Intracalcarine cortex left		
	496		77	Cuneal Cortex Right		
	437		9	Lateral occipital cortex, Superior division left		
	421		81	Cuneal cortex left		
	158		3	Precuneous cortex		
	98		69	Supracalcarine cortex		
	97		5	Lateral occipital cortex, inferior division Left		
	47		5	Occipital fusiform gyrus left		
	42		2	Occipital pole left		
	34		3	Cerebelum 6 Left		
	32		44	Supracalcarine cortex left		
	21		1	Lateral occipital cortex, inferior division right		
	18		1	Occipital Pole Right		
	7		1	Occipital fusiform gyrus right		
	5		0	Cerebelum crus1 left		
	4		0	Cerebelum 6 right		
	2		1	Vermis 6		
	1096		0	Not labeled		
Cluster 2	130		5	Occipital pole left		
Cluster 3	68		3	Lateral occipital cortex, inferior division right		
Cluster 4	14		1	Thalamus right		
	1		0	Subcallosal cortex		
	1		0	Caudate right		
	27		0	Not labeled		
Cluster 5	36		1	Occipital pole right		
	1		0	Not labeled		

Statistical thresholds: Voxel-threshold: *p* < 0.0002 p-uncorrected; cluster-threshold: *p* < 0.05 cluster-size p-FDR corrected; F(10,490) = > 3.41; k = > 37.

**TABLE 6:** Identified clusters from the Multi-voxel Pattern Analysis (MVPA) for the contrast CS2 vs. HS2.

Cluster	Coordinates (x,y,z)			k	p-FDR	p-unc
1	−10	−80	42	4569	<0.000001	<0.000001
2	54	−24	36	402	<0.000001	<0.000001
3	28	−92	4	183	0.000241	0.000014
4	36	32	26	78	0.023102	0.001777
**Anatomical coverage**						
	**Voxel count**		**% coverage**	**Region**		
Cluster 1	754		13	Precuneous cortex		
	481		93	Cuneal cortex left		
	471		73	Intracalcarine cortex left		
	463		72	Cuneal cortex right		
	462		10	Lateral occipital cortex, superior division Right		
	351		7	Lateral occipital cortex, superior division Left		
	300		40	Intracalcarine cortex right		
	250		16	Lingual gyrus left		
	115		7	Lingual gyrus right		
	63		44	Supracalcarine cortex right		
	50		68	Supracalcarine cortex left		
	12		0	Occipital pole right		
	9		0	Lateral occipital cortex, inferior division Right		
	2		0	Cingulate gyrus, posterior division		
	1		0	Temporal occipital fusiform cortex left		
	1		0	Occipital pole left		
	784		0	Not labeled		
Cluster 2	233		7	Postcentral gyrus right		
	76		9	Supramarginal gyrus, anterior division Right		
	49		1	Precentral gyrus right		
	29		2	Supramarginal gyrus, posterior division Right		
	7		0	Superior parietal lobule right		
	8		0	Not labeled		
Cluster 3	111		4	Occipital pole right		
	24		1	Lateral occipital cortex, inferior division Right		
	21		2	Occipital fusiform gyrus right		
	27		0	Not labeled		
Cluster 4	34		1	Middle frontal gyrus right		
	16		0	Frontal pole right		
	28		0	Not labeled		

Statistical thresholds: Voxel-threshold: *p* < 0.00025 p-uncorrected; cluster-threshold: *p* < 0.05 cluster-size p-FDR corrected; F(10,490) = > 3.41; *k* = > 78.

**TABLE 7:** Identified clusters from the Multi-voxel Pattern Analysis (MVPA) for the contrast HS1 vs. HS2.

Cluster	Coordinates (x,y,z)			k	p-FDR	p-unc
1	−54	−42	6	354	<0.000001	<0.000001
2	0	−82	26	166	0.000002	<0.000001
3	12	−70	20	129	0.000014	0.000002
4	54	−28	8	126	0.000014	0.000002
5	−14	−62	−6	109	0.000037	0.000008
6	22	−52	−10	32	0.021573	0.005628
**Anatomical coverage**						
	**Voxel count**		**% coverage**	**Region**		
Cluster 1	74		19	Superior temporal gyrus, posterior division Left		
	72		7	Supramarginal Gyrus, posterior division Left		
	49		6	Middle temporal gyrus, temporo-occipital part left		
	7		1	Middle temporal gyrus, posterior division Left		
	3		1	Planum temporale left		
	149		0	Not labeled		
Cluster 2	69		13	Cuneal cortex left		
	64		10	Cuneal cortex right		
	4		1	Intracalcarine cortex left		
	2		3	Supracalcarine cortex left		
	27		0	Not labeled		
Cluster 3	28		0	Precuneous cortex		
	24		4	Cuneal cortex right		
	21		3	Intracalcarine cortex left		
	7		1	Intracalcarine cortex right		
	7		5	Supracalcarine cortex right		
	2		3	Supracalcarine cortex left		
	40		0	Not labeled		
Cluster 4	65		15	Planum temporale right		
	1		0	Supramarginal gyrus, posterior division Right		
	60		0	Not labeled		
Cluster 5	109		7	Lingual gyrus left		
Cluster 6	28		2	Lingual gyrus right		
	4		0	Temporal occipital fusiform cortex right		

Statistical thresholds: Voxel-threshold: *p* < 0.00025 p-uncorrected; cluster-threshold: *p* < 0.05 cluster-size p-FDR corrected; F(10,490) = > 3.41; *k* = > 32.

The comparison control state 1 vs. control state 2 (CS1 vs. CS2), did not reveal any significant clusters and is thus not discussed further.

**FIGURE 3:**
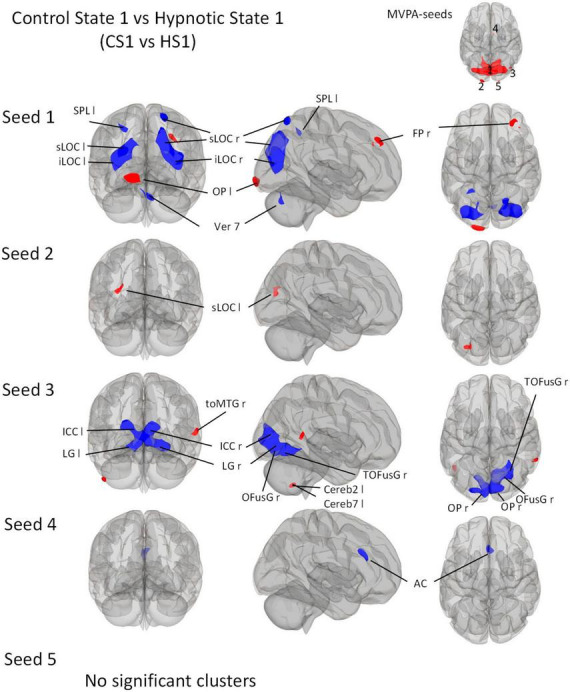
*Post-Hoc* seed-to-voxel analyses using the five MVPA-clusters from the comparison CS1 vs. HS1 as seeds. Voxel height threshold of *p* < 0.0002 (Bonferroni corrected for the five *post-hoc* analyses: *p* = 0.001/5) and cluster size threshold of *p* < 0.05 FDR were used as statistical thresholds. SPL, superior parietal lobule; sLOC, superior lateral occipital cortex; iLOC, inferior lateral occipital cortex; OP, occipital pole; Ver 7, cerebellar vermis 7; FP, Frontal Pole; toMTG, medial temporal gyrus, temporo-occipital part; LG, lingual gyrus; ICC, intracalcarine cortex; OFusG, occipital fusiform gyrus; Cereb, cerebellum; TOFusG, temporal occipital fusiform gyrus; AC, anterior cingulate cortex.

**FIGURE 4:**
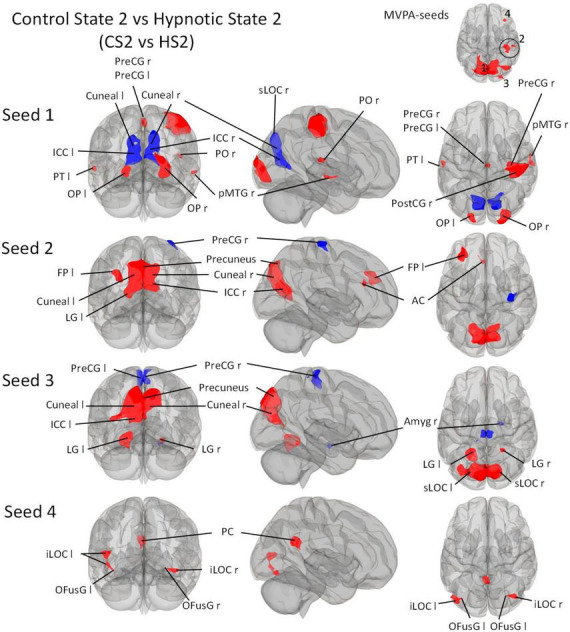
*Post-Hoc* seed-to-voxel analyses using the four MVPA-clusters from the comparison CS2 vs. HS2 as seeds. Voxel height threshold of *p* < 0.00025 (bonferroni corrected for the five *post-hoc* analyses: *p* = 0.001/4) and cluster size threshold of *p* < 0.05 FDR were used as statistical thresholds. SPL, superior parietal lobule; sLOC, superior lateral occipital cortex; iLOC, inferior lateral occipital cortex; OP, occipital pole; Ver 7, cerebellar vermis 7; FP, Frontal Pole; pMTG, middle temporal gyrus, posterior division; LG, lingual gyrus; ICC, intracalcarine cortex; OFusG, occipital fusiform gyrus; AC, anterior cingulate cortex; PC, posterior cingulate cortex, cuneal, cuneal cortex; preCG, precentral gyrus; postCG, postcentral gyrus; PT, planum temporale; Amyg, Amygdala.

**FIGURE 5:**
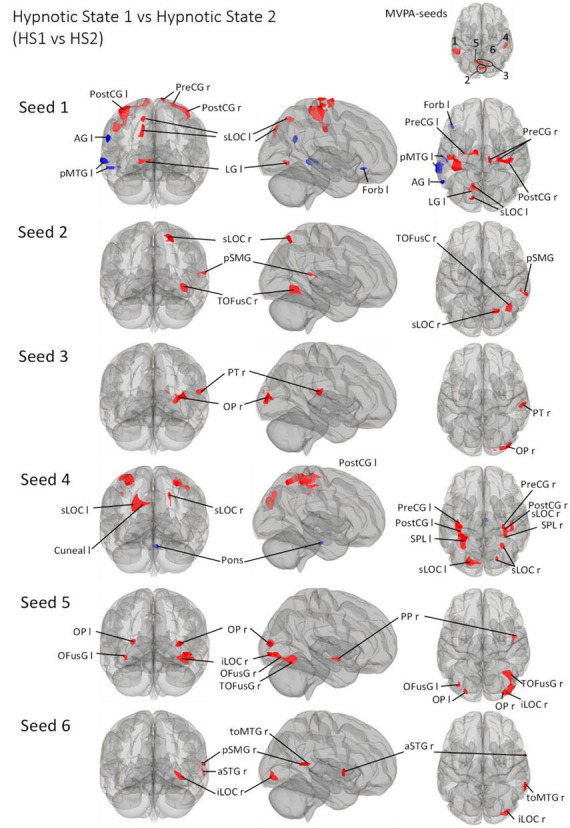
*Post- Hoc* seed-to-voxel analyses using the four MVPA-clusters from the comparison HS1 vs. HS2 as seeds. Voxel height threshold of *p* < 0.000167 (bonferroni corrected for the five *post-hoc* analyses: *p* = 0.001/6) and cluster size threshold of *p* < 0.05 FDR were used as statistical thresholds. SPL, superior parietal lobule; sLOC, superior lateral occipital cortex; iLOC, inferior lateral occipital cortex; OP, occipital pole; FP, Frontal Pole; pMTG, middle temporal gyrus, posterior division; LG, lingual gyrus; ICC, intracalcarine cortex; OFusG, occipital fusiform gyrus; TOFusG, temporal occipital fusiform gyrus; cuneal, cuneal cortex; preCG, precentral gyrus; postCG, postcentral gyrus; PT, planum temporale; Amyg, Amygdala; AG, angular gyrus; toMTG, medial temporal gyrus, temporo-occipital part; PP, planum polare; SPL, superior parietal lobule; aSTG, anterior superior temporal gyrus; pSTG, posterior superior temporal gyrus; pSMG, posterior supramarginal gyrus.

We then describe all *post-hoc* seed-to-voxel analyses of each of the identified MVPA clusters. These are illustrated in Figures 3–5.

#### 3.3.1 MVPA - Control state 1 vs. Hypnotic state 1

The comparison between Control State 1 and Hypnotic State 1 (CS1 vs. HS1, Figure 2 and Table 5) revealed five clusters. Four of these were mainly located in the parieto-occipito-temporal cortex and one in the anterior part of the right thalamus (cluster 4).

Of the occipital clusters, the largest one was medially located posterior to and along most of the parieto-occipital sulci of both hemispheres, reaching down to the bilateral lingual and fusiform gyri (cluster 1). This cluster further extended to dorsal components of the cerebellum. Two clusters were located at the occipital poles (cluster 2, 5). The last cluster encompassed the inferior part of the right lateral occipital cortex (cluster 3).

#### 3.3.2 MVPA - Control state 2 vs. Hypnotic state 2

The comparison CS2 vs. HS2 (Figure 2 and Table 6) identified four clusters, two of which were mainly located in the occipital cortices (cluster 1, 3).

The dominant cluster is very similar in localization to the largest cluster from the contrast CS1 vs. HS1, although having a smaller cluster size of approx. 23% less voxels (cluster 1). On par with the main cluster from the CS1 vs. HS1 contrast, it also was medially located posterior to the parieto-occipital sulcus of both hemispheres reaching the lingual gyri. In comparison to the main cluster of contrast CS1 vs. HS1, this cluster encompassed larger parts of the right superior lateral occipital cortex.

The other occipital cluster was mainly located at the right occipital pole (cluster 3).

Furthermore, the MVPA analysis resulted in the identification of two additional clusters, one located in the right postcentral gyrus (cluster 2) and one in the right middle frontal gyrus (cluster 4).

#### 3.3.3 MVPA - Hypnotic state 1 vs. Hypnotic state 2

The MVPA-analysis revealed six clusters for the contrast HS1 vs. HS2 (Figure 2 and Table 7). Compared to the previous contrasts, the largest cluster was not located in the occipital cortex. It was located at the left temporo-parieto-occipital junction, including the posterior superior temporal and posterior supramarginal gyrus, the temporo-occipital part of the middle temporal gyrus and the planum temporale (1). A second cluster was similarly situated in the other hemisphere, encompassing the right planum temporale and posterior supramarginal gyrus (4).

A group of four clusters were all closely located at the medial occipital cortex (2, 3, 5, 6) encompassing the lingual gyri, precuneous, bilateral cuneal, intracalcarine- and supracalcarine cortices.

#### 3.3.4 *Post-Hoc* seed-to-voxel results

##### 3.3.4.1 Control state 1 vs. Hypnotic state 1

Seed 1 correlated with seven significant clusters, five of which negatively (Figure 3). The negatively correlated clusters comprised large proportions of the lateral occipital cortex and some areas of the dorsal parietal cortex. A negatively correlated cluster was found in the cerebellar vermis.

In addition, the analysis revealed two positively correlated clusters, one located in the left occipital pole, and one in the right frontal pole.

Seed 2 was positively correlated with a single cluster, mainly located on the left lateral occipital cortex.

Seed 3 was shown to be correlated to three clusters. The largest one was negatively correlated and mostly encompassed structures of the ventro-posterior parts of the occipital cortices bilaterally. A positively correlated cluster was located in the left cerebellum, another at the right medial temporal gyrus.

Seed 4 showed negative correlations to one cluster located in the anterior cingulate cortex bilaterally.

For the *post-hoc* analysis of MVPA-seed 5, no significant clusters were identified.

##### 3.3.4.2 Hypnotic state 2 vs. Control state 2

Seed 1 from the contrast CS2 vs. HS2 was shown to be correlated with eight clusters (Figure 4). The largest cluster was negatively correlated and was medially and bilaterally located in the occipital cortex posterior to the parieto-occipital sulci. Two clusters were located on each occipital pole and one predominantly in the medial right precentral gyrus. A large cluster was localized on the right pre- and postcentral gyri. Two clusters were located on the temporal poles, one on the left planum temporale, and the other in the posterior middle temporal gyrus. One cluster was located in the right parietal operculum.

Seed 2 correlated with four clusters: The main cluster was positively correlated and situated in the occipital cortex posterior to the parieto-occipital sulci. The *post-hoc* analysis revealed two additional correlated clusters: One located in the left frontal pole, and one in the left anterior cingulate cortex. One negatively correlated cluster was found in the right precentral gyrus.

Seed 3 revealed functional connectivity to five clusters. Three were positively correlated to seed 2, all located in the occipital cortex. The largest was situated medially and adjacent to the occipital cortex posterior to the parieto-occipital sulci. The other two clusters encompassed the lingual gyri. One of the negatively associated clusters were located in the right Amygdala, the other encompassed the right and left precentral gyrus medially.

Seed 4 was positively correlated with three clusters: One in the posterior cingulate cortex bilaterally, and two on each lateral occipital cortex, both extending to the occipital fusiform gyri.

##### 3.3.4.3 Hypnotic state 1 vs. Hypnotic state 2

Seed 1 was shown to be functionally connected to a total of ten clusters (Figure 5), categorizable into three groups: Left fronto-temporo-parietal group, parieto-occipital group and the somatosensory group. The left fronto-temporo-parietal group consisted of four clusters, all negatively correlated to seed 1 for the defined contrast. These clusters encompassed brain areas belonging to the middle and superior temporal gyrus, angular and superior lateral occipital cortex, and frontal orbital cortex. The parieto-occipital group contained three clusters in the left lingual gyrus, left lateral occipital cortex, precuneus and cuneal cortex. The somatosensory group comprised three different clusters located in bilateral pre- and postcentral gyri ranging to the superior parietal lobule and superior frontal gyrus.

Seed 2 was positively correlated with three clusters, all located in the right hemisphere. The largest cluster was medially located in the occipital cortex, mainly in large parts of the right fusiform gyrus. The second cluster was located in the superior lateral occipital cortex. The last cluster was located at the temporo-parieto-occipital junction, in the posterior supramarginal and angular gyrus.

Seed 3 was positively correlated with two clusters in the right hemisphere, one at the right occipital pole spreading to the lateral occipital cortex, another at the right planum polare, parietal operculum and supramarginal gyrus.

Seed 4 was correlated to a total of six clusters, one of which was negatively correlated and located in the anterior pons. Two of the five positively correlated clusters were located at the pre-and postcentral gyri, encompassing the supramarginal gyri and superior parietal lobules. The left cluster also spread to the lateral occipital cortex. The other three clusters encompassed the superior lateral occipital cortices on both hemispheres, differing in their spread to cuneal, precuneus cortex and superior parietal lobule.

Seed 5 exhibited connectivity to five clusters, all positively correlated. One cluster was located at the right planum polare, the rest encompassed bilateral occipital poles and fusiform gyri, with larger cluster sizes on the right hemisphere spreading to the right inferior lateral occipital cortex.

Also, seed 6 revealed only positively correlated connectivity patterns to four areas (lateralized to the right hemisphere). Those areas covering anterior superior temporal gyrus, the medial temporal gyrus, temporo-occipital part, the inferior lateral occipital cortex as well as the posterior supramarginal gyrus.

## 4 Discussion

We investigated alterations in neural connectivity associated with hypnotic depth in 50 hypnosis-experienced healthy participants. The experimental approach differs significantly from other neuroimaging hypnosis studies. The most obvious difference relates to the fact that distinct control conditions were applied, namely content matched control texts for both hypnosis texts, which is not simply a so-called resting condition (Varga et al., 2017). This was possible since our hypnosis method is rigorously standardized and therefore all participants were hypnotized with exactly the same words. This is in contrast to a number of published studies, since participants are frequently guided into a hypnotic state *inter alia* using elements of personal content such as pleasant autobiographical memories, pleasant visual imagery, personal special/safe places (Maquet et al., 1999; McGeown et al., 2009, 2015; Demertzi et al., 2011; Deeley et al., 2012; Jiang et al., 2017)

Our method, however, aimed at a purely intrinsic/neutral hypnosis, hence a state of physical and mental relaxation, reached with minimal and neutral suggestions. We included participants highly familiar with this hypnosis inductions and depths. This strategy allowed to compare the hypnosis experiences inside vs. outside the scanner. The post-MRI questionnaire confirmed that the hypnotic state in the scanner was comparable to previously experienced states outside the scanner (Table 3).

Another feature of this study is the conducted fMRI analysis. From a behavioral viewpoint, it is generally accepted that hypnosis induces a wide range of sensations and experiences, some of these phenomena are very personal, some are comparable across participants (Zahedi and Sommer, 2021). This is one of the reasons why a scientific investigation of the hypnotic state remains a challenge (Posner, 1994, 2012; Rainville et al., 2002; Oakley and Halligan, 2013; Tagliazucchi et al., 2016; Boly et al., 2017; Northoff and Huang, 2017; Siclari et al., 2017; Storm et al., 2017; Terhune et al., 2017).

The diverse phenomena induced by hypnosis likely depend on the functional recruitment of multiple brain areas and are hardly manageable by single regions alone (Faymonville et al., 2006; Landry and Raz, 2015). This is supported by previous results showing that the neural effects of hypnosis can only be insufficiently explained by the activation of known brain network topology or specific single brain area activation (Landry and Raz, 2015; Landry et al., 2017). Also concepts focusing exclusively on top-down or bottom-up mechanisms do not seem comprehensive enough to adequately describe the neural processing underlying the phenomenal experiences of hypnosis (Jiang et al., 2017; Terhune et al., 2017; Zahedi and Sommer, 2021).

In order to adequately address this, we opted for a multi-voxel-pattern-analysis (MVPA). This method identifies voxels with altered connectivity to all other voxels of the brain in a data-driven and hypothesis-free way. This procedure is based on the latest methodological recommendations (Arnold Anteraper et al., 2019) and on the fact that no overarching neural correlate of hypnosis is currently defined (Landry et al., 2017).

Our study yielded robust results regarding hypnosis-induced changes when directly comparing the hypnosis states with the control states (CS1 vs. HS1 and CS2 vs. HS2) showing a fairly similar functional connectome pattern (Figures 3–5 and Tables 5, 6). The study further revealed - preliminary - evidence of hypnosis depth-dependent functional connectome changes (HS1 vs. HS2; Figure 5 and Table 7). These results are discussed in detail below.

### 4.1 Hypnosis states vs. Control states

The first analysis focused on the contrasts between both hypnotic states and their corresponding control states. The MVPA based analysis used for this purpose revealed that neural regulation mechanisms underlying the induced hypnosis states (reflected via the comparisons CS1 vs. HS1 and CS2 vs. HS2) are located within mainly parieto-occipital-temporal areas (Figure 2 and Tables 5, 6). It is worth mentioning that an MVPA reveals only areas characterized by changes in connectivity to all brain voxels, thus not reflecting any connectivity increase or decrease explained in detail in Material and Methods and respective literature (Whitfield-Gabrieli and Nieto-Castanon, 2012; Nieto-Castanon, 2020, 2022). Therefore, the focus of the discussion is on the corresponding seed-to-voxel analysis.

In contrast to the MVPA, which reveals a fairly similar connectivity pattern concerning CS1 vs. HS1 and CS2 vs. HS2 (Figure 2), the seed-to-voxel analysis shows more distinction in connectivity patterns (Figures 3, 4).

In general, the seed-to-voxel analysis showed that the comparison CS1 vs. HS1 is accompanied by a decrease in connectivity between most seeds and cortical brain regions (Figure 3). An exception is seed 1, which shows an increase in connectivity with OP1 l and FP r, as well as seed 3 with toMTG r.

The seed-to-voxel comparison of CS2 vs. HS2 is more heterogeneous (Figure 4). Starting from seed 1, a widespread connectivity decrease is observed, which affects both cuneal (Cuneal l/r) and ICC (ICC l/r) regions as well as a large fraction of the sLOC r. At the same time, however, a connectivity increase between seed 3 and MTG r is observed. Seed 1 also shows an increase in connectivity with OP l/r, PreCG l/r located medially in the interhemispheric gap, a very large area of PreCG r and PostCG r located mainly on the sensory and motor parts of the hand area, as well as small parts of PT l, PO r and pMTG r.

From a functional large network point of view, the areas covered by these main clusters incorporate the medial, occipital and lateral vision (Kalcher et al., 2012) as well as the higher order visual networks (Hutchison and Everling, 2012). These networks are part of reliably demonstrated configurations across resting state investigations and thought to (co)-organize different aspects regarding visual and spatial awareness, including visual and multimodal imagination, guidance of action as well as static and moving object recognition (Hutchison et al., 2013).

More recent studies, however, show “visual-network” contributions in patient populations not directly expected, as for example in patients suffering from chronic low back pain (cLBP). They demonstrated that cLBP patients show enhanced functional connectivity between the visual network and somatosensory/motor areas (Shen et al., 2019). The authors interpret those rather unexpected findings with adaptation and self-adjustment mechanisms due to cross-modal interactions between the visual and other networks often involved in processing cLBP (somatosensory/motor/attention/salient).

Our study furthermore shows that several cortical networks associated with altered states of consciousness may also contribute to the hypnotic state. Interestingly, some of the recent work is neuro-connectomically consistent with the results we found. Areas around a parieto-occipital cluster seem to be particularly prominently involved. In this context, we sometimes speak of the “posterior hot zone theory of consciousness” and are described to regulate a broad range of functions associated with altered conscious states (Crone et al., 2011; Bor, 2012; Heine et al., 2012; Sarasso et al., 2015; Koch et al., 2016a,b; Sato et al., 2016; Boly et al., 2017; Siclari et al., 2017). Dreaming is considered a change in consciousness and the Siclari group conducted an elegant study by investigating cortical network changes arising while people are dreaming using a serial awakening model (Siclari et al., 2017). They opted for an EEG approach since it is easier to waken participants during EEG recordings than during fMRI. They woke the participants several times during sleep and asked them if they could remember a dream and if so, what specifically they remembered. The main focus was on thought-like or explicit sensory experiences. EEG is to be interpreted rather cautiously with regard to activation localization. Nevertheless, the results show a relatively clear picture in the direction of parieto-occipital activation patterns while participants were dreaming. Specifically, when there was a decrease in low-frequency EEG patterns (1−4°Hz), participants remembered a dream, whereas when there was an increase, they did not.

In a recent EEG based modelling study, Ihalainen and colleagues found comparable results in propofpol-induced loss of consciousness (Ihalainen et al., 2021). The focus of this study was on the known neural networks, the DMN, SAL, and CEN. Methodically, a dynamic causal model (DCM) was calculated, which is a slightly different approach than ours and that of the Siclari group. Also, pharmacological sedation cannot really be compared to hypnosis. Therefore, we would also like to discuss this study cautiously in our context but would like to point out the strong embedding of parieto-occipital regions found to be involved here as well. Importantly, fronto-parietal connectivity patterns were also found, which is not the case in our study.

In our study, the lingual gyrus was involved in altered network configurations (this area is embedded in the posterior hot zone). This result was prominent in all statistical comparisons (Figures 3–5 and Tables 5–7 and therefore supports the core finding of the Landry et al. review (they found this structure as the lowest common denominator, meaning that “hypnotic responses correlate most robustly with activation in this area”). Landry and colleagues argue with two possible explanations regarding lingual gyrus function to co-regulate hypnosis (and failing to confirm their lead hypothesis regarding involvement of DMN, SN and CEN). On the one hand, they discuss an “intrinsic component of hypnosis” linked to mental imagery. On the other hand, they hypothesize that lingual activity results from “suggestion specific effects” due to visually compounded suggestions in order to induce hypnosis (Landry et al., 2017). However, only two of their reviewed publications aimed explicitly at inducing hypnosis via visualization techniques (Maquet et al., 1999; McGeown et al., 2012), whereas the other three reports sought to induce non-visual effects such as hand-paralysis (Cojan et al., 2009; Burgmer et al., 2013) or a non-specific suggestion guided hypnosis, focusing on deep mental relaxation (Rainville et al., 2002). It should be noted that this last study was predominantly an investigation of cortical pain mechanisms. The participants placed one hand in either pleasantly warm or painfully hot water (it was tested whether hypnosis influenced painful/painless sensations depending on water temperature). Interestingly, they revealed no effect of hypnosis on this variable of interest.

Interestingly, the temporo-parieto-occipital clusters found in our analysis also include several areas linked with cortical regions processing somatosensory and somaesthetic information originating from cutaneous and proprioceptive senses (Peyron et al., 2000). This fits into a more general interpretation regarding hypnosis effects as a holistically altered phenomenon of consciousness, associated with changes in different somatosensory/somaesthetic domains as well as an altered sense of agency manifested in semi-automatic, effortless and involuntary responses.

Interestingly, the data driven MVPA related with CS1 vs. HS1 revealed also connectivity changes in the cerebellum (Cluster 1, Table 5) and in the thalamus (Cluster 4, Table 5). Both, cerebellum and thalamus, are involved in different motor and perceptually discriminatory mechanisms (Barlow, 2002; Camarillo et al., 2012). It could therefore be hypothesized that these two subareas are involved in a basic co-regulation of the reported primarily physical relaxation in HS1. However, it is also important to mention that the effects seen in the cerebellum could be a spill-over effect from the large parieto-occipital cluster due to the spatial proximity and thus not representing a physiological connectivity alteration in the cerebellar vermis.

The deep physical and mental relaxation in HS2 is accompanied by perceptions of experienced bodily distortions, either of distinct body areas, or as a dissolution of body boundaries (sense of agency). In the debriefing, volunteers often reported that the upper extremities feel different, sometimes the hands are “felt” larger, or the arms are stretched away from the body, which - in realis - is of course not possible due to the tight conditions in the MR scanner. Apparently, modified coupling mechanisms of cortical somatosensory/sensorimotor integration systems takes place in the induced hypnotic state HS2, as is frequently reported in LSD (and other drugs) induced altered states of consciousness (Preller et al., 2019).

These somaesthetic changes are not uncommon under hypnosis (Zahedi and Sommer, 2021). We would like to point out that we are currently not able to clearly assign these descriptions to a corresponding connectomic pattern (due to the experimental setting). For this, the temporal dimension would have to be considered more precisely, i.e., we would have to know, when such a somaesthetic phenomenon occurs in order to calculate the associated connectomic correlate.

In line with this, an interesting result of the MVPA is cluster 2 in the contrast CS2 vs. HS2 showing significant connectivity changes in subdivisions of the right postcentral gyrus, right anterior and posterior supramarginal gyrus, the precentral gyrus and a small area covering right superior parietal lobule divisions (Figure 4 and Table 6). Hypnosis condition HS1 was not accompanied by such a clear connectivity change in somatosensory related areas (Figure 3 and Table 5). The reported profound changes in somatosensory experiences could thus be associated with the observed seed-to-voxel connectivity pattern of CS2 vs. HS2, reflected with significant connectivity increases in seed 1 with a right lateralized large cluster including pre- and postcentral subunits (PreCG r/PostCG r), together with a small subdivision within the posterior middle temporal gyrus (pMTG). Together with a decreased connectivity of seed 2 with PreCG r and seed 3 with bilateral PreCG subdivisions (PreCG r/PreCG l), this pattern may indicate that the altered somatosensory/somaesthetic perception characteristics are neurally recruited via this connectivity architecture (Figure 4 and Table 6). Further, complex signal/processing dynamics of the deep hypnotic state HS2 might be indicated in Figure 4. Seeds 1-3 interact with basically identical areas within a parieto-occipital cluster, but with different connectivity weightings (increase in red, decrease in blue). These comparisons refer to the respective contrasts between the hypnosis and control conditions, thus CS1 vs. HS1 and CS2 vs. HS2. It is worth mentioning that from a statistical perspective, these specific comparisons do not answer the question whether HS1 differs from HS2. Therefore, in order to make a statistically valid statement, the two hypnotic states must be directly contrasted. These aspects will be discussed in the next section.

In sum, based on the MVPA, we can show that the neural correlates of hypnotic states HS1 and HS2 strongly overlap when compared to the respective control states CS1 and CS2 (Figure 2). This is particularly true for the main cluster covering the following areas: Left/right cuneal cortex, left/right intracalcarine cortex, left/right inferior division of the lateral occipital cortex, left/right lingual gyrus, left/right occipital pole, the precuneous and left/right supracalcarine cortex are all structures involved in coding the transition from normal state (CS1/CS2) to the two hypnosis states (HS1/HS2). However, the main clusters of both comparisons also differ, albeit subtly. For instance, only HS1 involves the small aspects of the cerebellum (Table 5, cluster 1).

In HS2, small parts of the posterior cingulum, the right lateral-occipital cortex (superior division) and the left fusiform cortex (temporo-occipital division) are involved (Figure 2 and Table 6). Regarding the other hypnotic state specific peculiarities, MVPA based connectivity alterations in HS1 demonstrates the right thalamus being involved, whereas HS2 shows a pattern incorporating the right lateralized pre- and postcentral gyri as well as anterior/posterior divisions of supramarginal gyri (Figure 2 and Tables 5, 6).

### 4.2 HS1 vs. HS2: Digging deeper

When comparing HS1 vs. HS2, i.e., assessing the effect of hypnosis depth, more subtle changes are observed, evident by the results of the MVPA analysis showing a set of smaller clusters which engage in the network dependent on hypnosis depth. This goes along with the pattern of MVPA as direct comparison of the two hypnotic conditions show that no distinct large parieto-occipital areas appear to be recruited connectomically (Figure 2).

As with the discussion of the hypnosis vs. control condition comparisons, we follow a network hypothesis. Specifically, this means that we do not aim at an isolated function-based interpretation on the basis of single areas which was also declared to be not useful following the recent large meta-analysis by the Landry group (Landry et al., 2017).

The seed-to-voxel analysis predominantly showed connectivity increases for all six seeds (Figure 5). Connectivity decreases are primarily observed for seed 1 lateralized to the left (AG l, pMTG l, Forb l) and seed 4 (Pons). Considering all other connectivity changes - all connectivity increases - it is interesting that HS1 vs. HS2 is associated with the recruitment of significantly smaller areas compared to CS1 vs. HS1/CS2 vs. HS2.

These results shows that it is very likely that different hypnosis depths indeed exist and are supported by changes in neural network configuration.

From a behavioral perspective, however, this result is not completely unexpected. Just consider the normal human sleep, respectively, the different sleep phases as the theoretical explanatory construct. Sleep is not hypnosis, but sleep behavior shows clearly differentiated sleep stages (or sleep depths). This means that the brain does not normally fall into a deep sleep but seems to gradually “sink deeper and deeper” via regulatory mechanisms that have not yet been fully clarified. These are now beyond debate - although not thoroughly understood - and have been shown in many clinical and basic science papers (Saper and Fuller, 2017). Sleep is frequently compared with hypnosis, although these phenomena are hardly comparable. What is true is that sleep can be interpreted as an altered state of consciousness, just like hypnosis or - to a certain degree - coma (Saper and Fuller, 2017; Bonin et al., 2021). And just like hypnosis, sleep is seen as a complex phenomenon that exists in reality and can be clearly observed, but for which the underlying neural regulatory mechanisms are propofol investigated. In terms of neuroimaging, it must be said that sleep is perhaps even more difficult to study than hypnosis. This is because people have to sleep in an MR scanner, or rather, one must assume that people go through their natural sleep cycles despite the untypical and rather unpleasant MR environment. In addition, they should not move, which is actually against the nature of sleep as several observations showed (Gvilia, 2010; Saper and Fuller, 2017; Siclari et al., 2017).Now, as we have seen, in our setting neural correlates of two hypnotic states do not differ too dramatically when compared against their corresponding control states (CS1 vs. HS1 and CS2 vs. HS2).

However, when contrasting the two states directly against each other, quite specific connectome patterns emerged.

Interestingly, the neuro-connectomic regulatory mechanisms that allow the volunteers to move from HS1 to HS2 seem to occur in smaller areas (compared with the contrasts CS1 vs. HS1 and CS2 vs. HS2, in which relatively large areas - centered parieto-occipally - are prominently involved).

If we focus on the somatosensory aspects, the connectivity changes between seed 1, 2 and 4 and regions of motor (PostCG l/r, pons), supplementary motor (pMTG l) and sensory (PreCG r/l) regulation are striking.

The induced very deep physical and mental relaxation is neuro-connectomically supplemented by changes in intracalcarine (ICC), lateral occipital (sLOC/iLOC), occipital (OP), lingual (LG l/LG r), and cuneal (Cuneal l) regions (Figure 5).

Furthermore, the embedding of fusiform (OFusG r/TOFusG r) subregions as well as the planum temporale (PT) and planum polare (PP), starting from seeds 5 and 6, seems to be of additional importance when participants are in HS2.

The different experiences in this area suggest that the connectomic changes in these areas are associated with the reported somaesthetic phenomena (resolution of the somatosensory boundary concept as described at the beginning of this chapter).

In sum, we observed a connectomic pattern different from the patterns observed when contrasting CS1 vs. HS1 and CS2 vs. HS2. Generally, the involved clusters when comparing HS1 vs. HS2 are significantly smaller. Interestingly, previously delineated lingual gyrus divisions are still prominent supporting again data summarized in Landry et al. (2017). In addition, this implies that the so-called posterior hot zone is involved in the neural regulation of hypnotic depth.

### 4.3 Physiological measures and questionnaires:

Physiological parameter heart rate and heart rate variability revealed no differences between hypnotic states and control states whereas respiration patterns differed significantly between CS1 vs. HS1 and CS2 vs. HS2 but not between HS1 and HS2 (Table 4).

Physiological responses and questionnaires served mainly to allow objective characterization of hypnosis states and are not distinctively discussed in this report. The subjective characterization of the hypnotic state is limited in this study. This limitation will be addressed in the EEG-study of the project.

## 5 Limitations

We picked our study population selectively, meaning all participants have extensive hypnosis experience, especially with the two investigated states HS1 and HS2. This had the advantage that we could ask people after the measurement whether they perceived the hypnosis inside and outside the MR scanner differently. At the same time, it must be interpreted here as a limitation as we cannot generalize our results to a hypnosis naïve population. Additionally, no hypnotic suggestibility was assessed as selection or control criterium. This approach differs from the existing literature thus impeding generalizability and comparability. Future studies with hypnosis-naïve participants should apply standard suggestibility assessments to clarify whether our results are generalizable.

Our data inform the discussion on different hypnotic states or depths. This concept is highly debated as no reliable independent markers had been discovered. Based on the questionnaires, we can assume that HS1 differs from HS2. Additionally, the MVPA and the seed-to-voxel calculations (purely data driven) supports the notion, that HS2 represents a different hypnotic state than HS1. However, also with respect to this result, a hypnosis-naïve population should be investigated. Please also note that the used control texts were not evaluated by means of quantitative methods such as inter-judge evaluations or natural language processing techniques. This should be considered in future studies.

Furthermore, it’s important to note that the test subjects may carry a certain degree of bias due to their training, a factor that is nearly impossible to eliminate entirely. However, it’s also crucial to recognize that it’s not typically feasible for individuals to intentionally alter brain response patterns to a degree that would result in the (remarkable) consistency of the functional neuro-connectome, as is evident in our data. This study is part of a larger project to better understand the neurobiological correlates of hypnosis. One of the aims is to investigate whether objective markers for hypnosis depth exist. Thus, with the present report, although we have possible indications toward two different hypnotic states, we are cautious with a final interpretation regarding this outcome. If this pattern is equally robust in the future studies of the project (MRS and EEG), we might be able to determine multimodal neurobiological markers possibly reflecting different hypnotic depths or states. Due to the EEG setting, we will further be able to report behavioral components of different hypnotic states which are not available in this study. Nevertheless, it’s necessary to investigate a hypnosis-naive population with regard to this possible outcome. In addition, this setting should be replicated at other locations and ideally also compared with other forms of hypnosis. A factor that is likely not to be underestimated is the fact that the participants were in an untypical situation with a supine position in the MRI scanner, as well as being exposed to the noise of the MRI scanner. We discussed this issue intensively before the project and under the given circumstances, it is not really satisfactorily solvable. However, to keep the noise level to a minimum, we used the specially designed headphones from MRConfon, Magdeburg, Germany. These are significantly more comfortable to wear and reduce noise exposure better than the devices typically used.

## 6 Conclusion and outlook

We showed that - in a specific experimental setting incorporating a hypnosis-experienced population, hypnosis can be reliably induced and measured using fMRI.

By following a data driven analysis approach, we observed functional network configurations that only partially correspond to the typically observed networks described in the literature (Hutchison et al., 2013). It seems more likely that the effect of hypnosis is associated with connectomical changes in areas that are also debated in the discussion of the neural correlates of consciousness (Delacour, 1997; Bayne et al., 2016; Boly et al., 2017). Interestingly, we partially support the findings of a recent large neuroimaging based meta-analysis, which elucidated that the neural correlate of hypnosis cannot be described by common neural network concepts (Landry et al., 2017). Furthermore, our data corroborate an interesting finding of this review article: the involvement of an area incorporating the lingual gyrus as an important brain area for in processing hypnosis, potentially as main hub.

Nevertheless, with this work we do not explain why and how hypnosis shows convincing results in specific clinical settings. We also cannot generalize the results shown here to other settings. Furthermore, it still needs to be investigated if the observed connectivity changes are specific for hypnosis or are also other mind-altering methods accompanied by similar connectome changes.

## Data availability statement

The original contributions presented in the study are included in the article/Supplementary material, further inquiries can be directed to the corresponding author.

## Ethics statement

The study involving humans was approved by the Kantonale Ethikkommission Zürich, Basec Nr 2018-00550. The study was conducted in accordance with the local legislation and institutional requirements. The participants provided their written informed consent to participate in this study.

## Author contributions

NM: Conceptualization, Formal analysis, Investigation, Methodology, Project administration, Supervision, Visualization, Writing – original draft, Writing – review & editing, Data curation, Funding acquisition, Resources, Software, Validation. PS: Investigation, Project administration, Supervision, Writing – review & editing, Resources. ES: Writing – review & editing. KP: Writing – review & editing. MB: Conceptualization, Data curation, Formal analysis, Funding acquisition, Investigation, Methodology, Project administration, Resources, Software, Supervision, Validation, Visualization, Writing – original draft, Writing – review & editing.

## Footnotes

1
http://www.fil.ion.ucl.ac.uk/spm/
