# Supplementary material for: Investigating functional brain connectivity patterns associated with two hypnotic states
Source: Front Hum Neurosci. 2023 Dec 19;17:1286336. doi: 10.3389/fnhum.2023.1286336 (PMC10773817; doi:10.3389/fnhum.2023.1286336)
Supplement: Supplementary file 1 [file Data_Sheet_1.PDF]

Control Texts for Project Hypnoscience

Control Condition 1 (CS1)

When breathing, air flows into the body through the mouth or the nose. When inhaled through the nose, the air is first cleaned, moisturized, and warmed by the tiny hairs and mucous membranes in the nose. Subsequently, the respiratory air passes through the throat, past the larynx and vocal cords, and into the trachea.

With regard to the eye muscles, they can be classified into inner and outer eye muscles based on their location and function. This includes the muscle that lifts the eyelid and the ones associated with the nictitating membrane found in some vertebrates.

The aim of all relaxation techniques is to induce a relaxation response, which manifests on a neural level as an activation of the parasympathetic nervous system and a weakening of the sympathetic nervous system.

The human voice is the sound produced by the vocal cords of a person and modulated in the oral, throat, and nasal cavities.

'Name of the test subject,' please indicate your mental presence by slightly lifting your index finger on your left hand... Wait until the finger on the left hand is raised

Closing and opening of the eyelids are referred to as eyelid closure or generally blinking. The closing movement occurs either voluntarily or involuntarily through the blink reflex, which fully closes the eyelids in response to certain stimuli.

The closing of the eyelid usually happens faster than the opening. Additionally, regular blinking ensures that the sensitive cornea, which plays a crucial role in light refraction and optimal vision, as well as the front part of the sclera, are adequately moistened with tears at all times, protecting them from drying out.

The frequency of blinking in humans is approximately 10-12 blinks per minute, with women blinking faster and more frequently than men. The closure of the eyelids occurs synchronously and is typically achieved through the interaction of the upper and lower lids.

The hand is the gripping organ of the upper extremities in primates. In humans and most primates, it is distinguished by the opposable thumb with the opponens pollicis muscle, enabling the pincer grasp. In other terrestrial vertebrates, the term "forelimb" is mostly used.

"Name of the test subject," please indicate your mental presence by slightly lifting your index finger on your left hand... Wait until the finger on the left hand is raised.

Relaxation techniques are practiced methods aimed at reducing physical and mental tension or arousal. On a physical level, muscle tone is reduced, reflex activity is diminished, peripheral blood vessels dilate, heart rate slows down, and arterial blood pressure decreases.

On a psychological level, relaxation response is associated with a sense of calmness, satisfaction, and well-being, as well as improved concentration and discernment of physical sensations.

Counting is an action performed to determine the number of elements in a finite set of objects of the same kind.

In mathematics, which formally studies numbers and their structure, the term encompasses various concepts. These concepts evolved as generalizations of existing intuitive notions of numbers, even though they may have little connection to the originally associated concepts of measurement.

Short pause of 5-10 seconds.

"Name of the test subject," please indicate your mental presence by slightly lifting your index finger on your left hand... Wait until the finger on the left hand is raised.

Natural numbers are the numbers used for counting: 1, 2, 3, 4, 5, 6, 7, 8, 9, 10, and so on. Depending on the definition, 0 (zero) may also be included as a natural number. The set of natural numbers, together with addition and multiplication, forms a mathematical structure known as a commutative semiring.

The set of natural numbers is abbreviated as N. In the widely used Unicode character encoding, it corresponds to the character with the codepoint U+2115.

It either includes positive integers (excluding 0) or non-negative integers (including 0). Silence refers to the perceived absence of sound or any noise, as well as motionlessness. Its colloquial intensification is known as complete silence or dead silence.

Measurement is the process of performing planned activities to obtain a quantitative statement about a measurement quantity by comparison with a unit.

"Name of the test subject," please indicate your mental presence by slightly lifting your index finger on your left hand... Wait until the finger on the left hand is raised.

Okay. Very good. We're about to begin now.

Control Condition 2 (CS2)

Very good, you're doing an excellent job. Let's continue.

A plane is a fundamental concept in geometry. In general, it refers to an infinitely extended, flat two-dimensional object. Infinitely extended and flat mean that any two points in the plane determine a line that lies entirely within the plane.

In classical geometry, such as in Euclid's Elements, the (Euclidean) plane - usually referred to with the definite article in this context - provides the framework for geometric investigations, such as constructions using a compass and straightedge.

An escalator (or moving staircase) is a means of transportation for people to overcome a vertical distance, where moving metal or (formerly) wooden segments form steps.

"Name of the test subject," please indicate your mental presence by slightly lifting your index finger on your left hand... Wait until the finger on the left hand is raised.

In image editing, layering technique is used to distribute elements of the image onto different layers. These layers can be individually edited and combined in various ways. This technique is a standard in image editing.

"Name of the test subject," please indicate your mental presence by slightly lifting your index finger on your left hand... Wait until the finger on the left hand is raised.

Since the plane is transparent like a sheet, the background image and the text appear to the viewer as a single image.

Layering technique is often used in advertising agencies. For example, one can overlay a background image, such as human skin, with a tiger skin or zebra pattern to achieve an effect of alteration. Collages can also be created using the layering technique.

"Name of the test subject," please indicate your mental presence by slightly lifting your index finger on your left hand... Wait until the finger on the left hand is raised.

Silence refers to the perceived absence of sound or any noise, as well as motionlessness.

Okay... we're starting with the measurement. Please don't fall asleep or go back into a state of hypnosis, but stay awake. Thank you

Hypnosis Texts for Project Hypnoscience

Hypnosis State 1 (HS1)

Breathe in deeply once now, hold your breath briefly... and as you exhale, you may close your eyes. Allow any superficial tension to leave your body. Relax your body as much as you can.

Now, focus on your eye muscles and relax all the muscles around your eyes until you simply can't open your eyes anymore. And when you are confident that you have relaxed your eye muscles to the point where they simply refuse to function, hold onto this relaxation and test your eyelids to be sure that THEY REALLY DON'T WORK ANYMORE. (Allow 3-4 seconds to test and then say:) Good, and stop testing.

Now, I want you to extend the same relaxation you feel in your eyes to your entire body. Let the same quality of relaxation flow through your entire body, from your head down to your toes.

You are fully concentrated on my voice. Every word I say helps you relax even more. Of course, you may perceive other sounds, perhaps voices, footsteps, the ventilation, or functioning devices. But none of that disturbs you. On the contrary, these background noises help you relax even more.

And now, we can deepen this relaxation even further, and here's how: In a moment, I will ask you to briefly open your eyes and then close them again. Once your eyes are closed, take it as a sign that you can relax your body even more. You just need to want it and allow it, and it will happen effortlessly.

Okay, open your eyes now... and close them again, and relax even deeper this time... good. Let every muscle in your body relax so much that as long as you want to hold onto this relaxation, no muscle will function anymore.

In a few seconds, I will ask you again to briefly open your eyes, and when you close them, you can simply let go even more, sink even deeper, completely loose and light. Okay, open your eyes now... and close them again, and relax even deeper this time... good, very good. Let all the muscles in your body relax completely, and as you deepen this relaxation, they will simply refuse to function.

In a moment, I will ask you to focus on your right or left hand. If you have followed my instructions so far, you will notice that your hand lies completely loose and relaxed, like a wet cloth. It may feel very light or perhaps quite heavy. However it feels, it's okay.

Good. Now, direct your attention to your hand and allow yourself to perceive this feeling, and now follow this feeling even deeper into this wonderful, deep relaxation. Very good. This is total physical relaxation. I want you to know that there are two types of relaxation. You can relax physically, and you can also relax mentally. You have already demonstrated that you can relax physically. Allow me to show you how to relax mentally, which is even easier and feels even better.

In a moment, I will audibly count backward from 100, because this is the key to your mental relaxation. With each number I say, you allow yourself to deepen your mental relaxation, let go even more. With each number I say, you let your mind relax even deeper than before.

And when you do this and I reach the number 98 - or maybe even before - you will be so relaxed that all the numbers that come after 98 will simply vanish from your mind. There are no numbers anymore. It doesn't matter to you, and you don't care if there are any left. Only you can make this happen; I can't do it for you. The numbers will disappear if you want them to. Start now with the thought that it will happen just like that, and it will be easy for you to push them out of your mind. I will say the first number now, 100, and as I do, allow your mind to relax even deeper, let go even more.

Hypnotist: 100... deeply relaxed... let them go, let them fade away, and simply relax even deeper...

Hypnotist: 99... deeply relaxed. You no longer need them, let them go. They will fade away. They will disappear if you want them to. And go deeper.

Hypnotist: 98... deeply relaxed. Let them all fade away. Banish them, let them disappear. You can do it. Very good. Short pause of 5-10 seconds. Deepening phase: Wonderful. In a moment, I will count from 10 down to 1. With each number I say, allow yourself to let go even more, to go even deeper. When I reach one or even before that, you will be even more deeply relaxed than before.

10... let yourself go, let yourself sink, deeper and deeper... relaxed. 9... with each exhale, simply let go even more. 8... 7... 6... 5... the deeper you go, the better it feels... 4... and the better it feels, the deeper you can go once again. 3... 2... 1... good. Very good.

Now, I will give you a brief moment of silence and stillness. In this moment of silence and stillness, allow yourself to sink even deeper. This moment of silence and stillness begins... now.

Soft, calm voice: Okay, wonderful. We will now begin the measurement in the state of deep somnambulism. It's important that you remain in somnambulism during the measurement. If you are still willing to listen to me and follow the instructions, show me by slightly lifting the index finger of your left hand... Wait until finger on the left hand rises.

Okay. Very good. We're about to start.

Hypnosis State 2 (HS)

Great, you're doing an excellent job. Let's continue.

I want to take you now to the deepest level of your personal relaxation, where you will feel sensational. To a place where all your worries, problems, concerns, and discomfort simply vanish. It's a wonderful place. And to reach this place, I will ask you to go three levels deeper than where you currently are. Let's call these levels, for the sake of clarity, Level A, Level B, and Level C.

To descend to Level A, all you have to do is double your current level of relaxation. To reach Level B, you double the relaxation you have in Level A. And to reach the deepest level, Level C, you simply double the relaxation of Level B.

Okay, let's begin. Imagine that you're standing at the top of your personal escalator. It can also be an elevator, a slide, a pathway—whatever you choose. In a moment, I will count from 1 to 3. When I reach 3, this escalator will take you down to Level A. Once you're there, your current level of relaxation will have doubled. However, you decide how long or short this escalator is. You can also determine the speed at which it descends.

When you reach Level A, let me know by simply lifting the index finger of your left hand slightly. Okay, here we go. 1 – 2 – 3... all the way down to Level A... deeper and deeper relaxed" (wait without saying anything until the participant raises their left index finger).

"Very good... Shortly, I will count from 1 to 3 again. Once I reach 3, your escalator will take you from Level A to Level B, where, once you arrive, your level of relaxation will have doubled once more. When you reach Level B, try to lift the index finger of your left hand slightly. If you follow my instructions carefully, once you're in Level B, you will notice that your hand is so relaxed that it has become extremely difficult to lift your index finger at all.

But that's exactly what we want. Lift your index finger despite the difficulty, so I know that you have arrived down there. Okay, let's continue. 1 – 2 – 3 – go all the way down to Level B... deeper and deeper relaxed..." (wait without saying anything until the participant raises their left index finger).

"Very good. You're doing an excellent job. Now we just need to go down one more level to Level C. This is the true foundation of total relaxation in this moment. A place where you feel extremely comfortable, simply good. A place where all your problems, whether physical or mental, have disappeared. Let it happen, allow it, and it will simply come naturally for you to reach that level.

Okay, once again, you're at the top of your escalator, looking down. I will count from 1 to 3 again, and then this escalator will take you all the way down to Level C, your deepest relaxation ever. Once you have reached Level C, let me know by gently lifting your left index finger. But don't try to help it. Stay relaxed and let it simply happen. And here we go: 1 - 2 - 3 - all the way down to Level C, your deepest relaxation ever, deeper and deeper and deeper, let go, drift away, surrender, deeper... and deeper... and deeper..." (wait without saying anything until the participant raises their left index finger).

Very good. Now I will give you a brief moment of silence and stillness. In this moment of silence and stillness, allow yourself to sink even deeper. If there is a Level D or even E for

you, go down there. Let it simply happen. The measurement will begin shortly, and you will remain in this deep state. This moment of silence and stillness starts now.

Wait for 30 - max. 60 seconds. Soft, calm voice: Okay... Here we go.
